# Advanced Chronic Kidney Disease with Low and Very Low GFR: Can a Low-Protein Diet Supplemented with Ketoanalogues Delay Dialysis?

**DOI:** 10.3390/nu12113358

**Published:** 2020-10-31

**Authors:** Chieh-Li Yen, Pei-Chun Fan, Cheng-Chia Lee, George Kuo, Kun-Hua Tu, Jia-Jin Chen, Tao-Han Lee, Hsiang-Hao Hsu, Ya-Chun Tian, Chih-Hsiang Chang

**Affiliations:** 1 Kidney Research Center, Department of Nephrology, Chang Gung Memorial Hospital, Linkou Branch, Taoyuan 33341, Taiwan; b9102087@yahoo.com.tw (C.-L.Y.); franwis1023@gmail.com (P.-C.F.); a12490@cgmh.org.tw (C.-C.L.); b92401107@gmail.com (G.K.); 8902086@cgmh.org.tw (K.-H.T.); Raymond110234@hotmail.com (J.-J.C.); kate0327@hotmail.com (T.-H.L.); hsianghao@gmail.com (H.-H.H.); dryctian@cgmh.org.tw (Y.-C.T.); 2 College of Medicine, Chang Gung University, Taoyuan 33341, Taiwan

**Keywords:** chronic kidney disease, Ketosteril, low-protein diet, dialysis, adverse events

## Abstract

Background: Previous studies have demonstrated that dietary therapy can delay the initiation of dialysis, but little research has investigated whether patients with very poor renal function would benefit from a dietary therapy. Methods: This study was performed by using the Chang Gung Research Database (CGRD), which is based on the largest medical system in Taiwan. Patients with estimated glomerular filtration rates (eGFR) < 15 mL/min/1.73 m^2^ between 2001 and 2015 with more than 3 months of low-protein diet supplemented with ketoanalogues (sLPD) were extracted (Ketosteril group). We then assigned five patients without any sLPD to match one patient of the Ketosteril group (comparison group). Both groups were followed up for 1 year for the initiation of dialysis and rates of major adverse cardiac and cerebrovascular events (MACCEs). Results: The Ketosteril group (*n* = 547), compared with the comparison group (*n* = 2735), exhibited a lower incidence of new-onset dialysis (40.2% vs. 44.4%, subdistribution hazard ratio (SHR): 0.80, 95% confidence interval (CI): 0.70–0.91) and MACCEs (3.7% vs. 5.9%, HR: 0.61, 95% CI: 0.38–0.97). The beneficial effect of an sLPD did not differ in patients with a baseline eGFR < 5 mL/min/1.73 m^2^. Conclusion: Even among patients with extremely low eGFR, sLPD treatment can safely delay the need for dialysis.

## 1. Introduction

The use of dietary therapy to treat chronic kidney disease (CKD) has been investigated for more than a century [1,2]. However, a long-term low-protein diet without any nutritional supplementation may lead to malnutrition, thus limiting its application [3]. It was not until the 1970s that Walser et al. first demonstrated that a very low-protein diet supplemented with ketoanalogues (sVLPD) could safely retard the progression of CKD without increasing its adverse effects [4]. Theoretically, while protein intake is restricted, essential amino acids (EAAs) ketoanalogues can be transaminated into corresponding EAAs [5]. Through this process, both sVLPDs (0.3–0.4 g/kg body weight per day) [6] and low-protein diets supplemented with ketoanalogues (sLPDs) (0.6 g/kg body weight per day) [7] can dampen the consequences of protein metabolism, such as the accumulation of nitrogen waste products, metabolic acidosis, and hyperphosphatemia, while maintaining the protein–energy balance. Indeed, several randomized controlled trials (RCTs) [8,9] and observational studies [10,11] have proved that sVLPDs and sLPDs can delay the initiation of dialysis and slightly retard the decline of renal function. Moreover, in those who can adhere to the treatment and frequent follow-up of nutritional status, dietary therapy has been shown not to result in adverse effects during treatment [12,13] or after dialysis initiation [10,14].

However, such previous research on dietary therapy has mostly enrolled patients with baseline estimated glomerular filtration rates (eGFR) of 10–30 mL/min/1.73 m^2^ [12,15]. There is little evidence to answer the question of whether patients with extremely poor renal function (eGFR < 10 mL/min/1.73 m^2^) should start dietary therapy if they have not received it before. This is a question worth addressing for at least two reasons. First, long-term protein restriction is unpleasant and difficult to follow for most patients. Prior studies have demonstrated that the adherence of dietary therapy is only approximately 50% [16]. In addition, the large number of daily Ketosteril tablets, which is the brand name of the common-use ketoanalogues, required (e.g., at least 12 tablets/day for a 60 kg adult undergoing sVLPDs) is a considerable burden for these patients. Thus, a large portion of patients may be unwilling to undergo dietary therapy until very close to commencing dialysis. Second, high-quality dietary therapy requires an experienced, professional treatment team including kidney specialists, nurses, and dietitians, which may not be available in all institutions. In many cases, CKD patients may be treated by a non-kidney specialist who is unfamiliar with dietary therapy, or may be treated in an institution without a dietary therapy group, and then may be finally transferred to a kidney specialist only for dialysis preparation. Therefore, whether dietary therapy should be started in these clinical scenarios warrants investigation. By using the dataset of Chang Gung Memorial Hospital system, which is the largest hospital network in Taiwan, this study aimed to determine the effects of dietary therapy among patients with extremely low eGFR.

## 2. Materials and Methods

### 2.1. Data Source

This was a retrospective cohort study utilizing the Chang Gung Research Database (CGRD). The CGRD is a de-identified database based on the electronic medical records of the Chang Gung Memorial Hospital system, which is currently the largest medical system in Taiwan, comprising 4 tertiary medical centers and 3 other teaching hospitals and covering more than 10% of all Taiwan’s annual medical services [17,18]. The CGRD contains the comprehensive medical records—including outpatient visits, inpatient orders, medication prescriptions, procedure interventions, laboratory data, and examination reports—of the entire Chang Gung Memorial Hospital systems. The identification of diseases in the CGRD is based on the International Classification of Diseases, 9th Revision, Clinical Modification (ICD-9-CM) for data before 2016 and ICD-10-CM for data thereafter. Data that could identify patients are encrypted and de-identified before being entered into the CGRD to protect their privacy, and its consistent data encryption enables medical information to be linked for research purposes. Thus, the need for informed consent for this study was waived by the Chang Gung Medical Foundation’s Institutional Review Board (approval number: 201800002B0).

### 2.2. Patient Selection and Study Design

**Figure 1:**
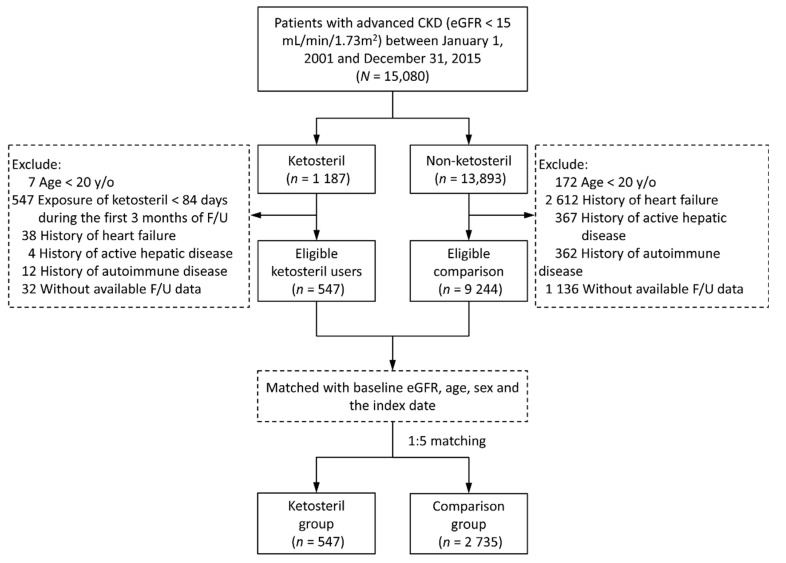
Flowchart for the process of inclusion and exclusion of study patients. CKD, chronic kidney disease; eGFR, estimated glomerular filtration rate; F/U, follow up; y/o, years old.

Before 2016, Taiwan’s National Health Insurance regulations allowed Ketosteril to be prescribed without copayment only for patients with serum creatinine > 6 mg/dL. In addition, these patients were required to adhere well to a low-protein diet and to visit a dietitian for consultation at least once. Because of these regulations, the use of Ketosteril prior to 2016 is a reasonable surrogate for sLPD. As illustrated in Figure 1, we extracted CGRD patients aged 20 years or more with eGFR < 15 mL/min/1.73 m^2^ who had not previously received renal replacement therapy between 2001 and 2015. The patients were divided into 2 groups according to the use of Ketosteril.

To reduce potential confounders, patients with a history of heart failure, active liver disease, or autoimmune disease were excluded irrespective of the use of Ketosteril. Patients who received Ketosteril for less than 84 days in the first 3 months after the initiation of Ketosteril treatment were excluded to ensure a high compliance population. The first prescription date of the Ketosteril treatment was defined as the index date. The remaining patients were classified into the non-Ketosteril comparison group. Afterward, to avoid immortal time bias, we randomly assigned the index date of 1 patient in the Ketosteril group to 5 patients in the comparison group through frequency matching with baseline eGFR, sex, and age.

### 2.3. Covariates

The covariates examined were age, sex, baseline comorbidities (hypertension, diabetes, hepatitis B virus infection, and hepatitis C virus infection), baseline laboratory data (eGFR (Modification of Diet in Renal Disease(MDRD)), urine albumin/creatinine ratio, urine protein/creatinine ratio, urea, uric acid, bicarbonate, calcium, and phosphate), and anti-hypertensive medications (angiotensin converting enzyme inhibitors (ACEi)/angiotensin II receptor blockers (ARB) and other anti-hypertensive agents). Baseline comorbidities were detected using 2 or more outpatient diagnoses or 1 or more inpatient diagnosis prior to the index date. Baseline laboratory data and medications were extracted from medical records for 90 days before or after the index date. When multiple laboratory data records existed, the 1 recorded most closely to the index date was used.

### 2.4. Outcomes

The primary outcome of this study was end stage renal disease (ESRD) requiring maintenance dialysis based on the clinical criteria of the treating nephrologist. The secondary outcomes were all-cause mortality, acute myocardial infarction (MI), ischemic stroke, and major adverse cardiac and cerebrovascular events (MACCEs) as well as infection-related hospitalization and heart failure hospitalization. The MACCEs were defined as a composite of all-cause mortality, acute MI, and ischemic stroke. These outcomes were identified according to the CGRD medical records. Acute MI, ischemic stroke, infection-related hospitalization, and heart failure hospitalization were identified based on the principle diagnosis of hospitalization or emergency room visit. The follow-up period lasted from the index date to the first occurrence of any study outcome, the date of dialysis initiation, the date of death, or the 365th date after the index date, whichever came first. The 1 year follow-up eGFR was calculated using the final serum creatinine recorded before the 365th date after the index date or using the serum creatinine recorded from the initiation of dialysis if new-onset ESRD came within one year of the index date.

### 2.5. Statistical Analysis

The baseline characteristics of the patient groups (Ketosteril vs. non-Ketosteril) were compared using the independent sample t-test for continuous variables or the chi-square test for categorical variables. The risk of time to a fatal event (i.e., all-cause mortality and MACCEs) between the groups was compared using the Cox proportional hazard model. The risk of other time to the event outcome (i.e., dialysis) between the groups was compared using the Fine and Gray subdistribution hazard model, which considered all-cause mortality a competing risk. We additionally adjusted hypertension, diabetes, ACEIs/ARBs, hepatitis C virus infection, calcium supplementation and vitamin D therapy in the survival analyses. The first three covariates (hypertension, diabetes and ACEIs/ARBs) were adjusted based on a clinical perspective. The later three covariates (hepatitis C virus infection, calcium supplementation and vitamin D therapy) were adjusted due to the significant difference between the two patient groups. Serum urea and serum bicarbonate were not adjusted because of the presence of missing values. Outcome dependency existed among patients within the same matching pair; therefore, the within-pair clustering of outcomes was accounted for by using a robust standard error. The change in eGFR level between baseline and 1 year follow-up was compared using the Mann–Whitney U-test. The median duration to dialysis (expressed in months) between the study groups was also compared using the Mann–Whitney U-test.

A two-sided *p* value of <0.05 was considered statistically significant, and no adjustment of multiple testing (multiplicity) was performed. All statistical analyses were performed using SAS version 9.4 (SAS Institute, Cary, NC, USA), including the PHREG procedure for survival analysis and the %CIF macro for generating the cumulative incidence function for the Fine and Gray subdistribution hazard testing.

## 3. Results

### 3.1. Patient Characteristics

A total of 3282 adult patients with advanced CKD (eGFR < 15 mL/min/1.73 m^2^) between 2001 and 2015 were extracted, of whom 547 patients had been on Ketosteril treatment for more than 3 months. Their demographics, comorbidities, medications, and baseline laboratory data are displayed in Table 1. Most patients in the two groups (Ketosteril group: 87.2%; non-Ketosteril group: 84.9%) had a baseline eGFR < 10 mL/min/1.73 m^2^. Approximately one-fifth of the cohort had extremely low eGFR levels (<5 mL/min/1.73 m^2^). The results showed that the Ketosteril group had more prescriptions for ACEi/ARBs, calcium supplementation, vitamin D therapy, higher prevalence of hepatitis C virus infection, higher serum urea, and bicarbonate than the non-Ketosteril group. The two study groups did not differ significantly in other characteristics (Table 1).

### 3.2. Outcomes during 1 Year Follow-Up

**Figure 2:**
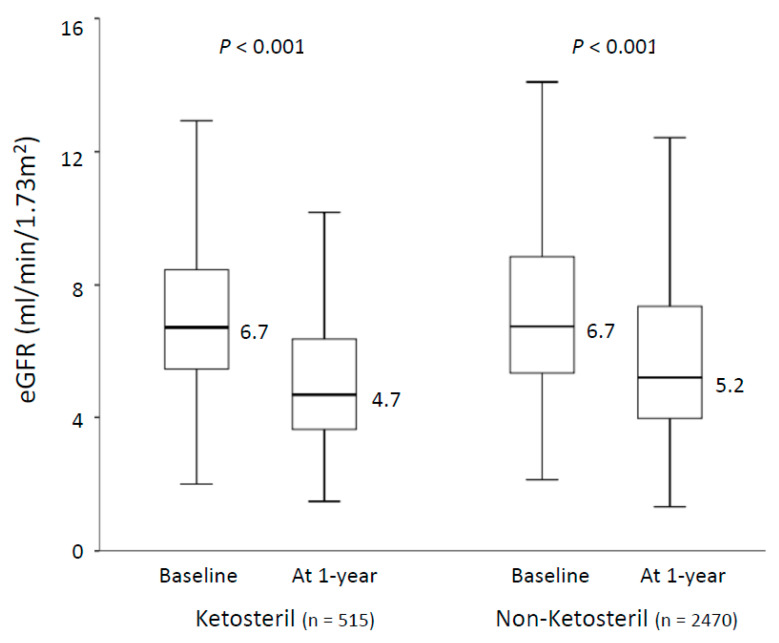
Mean eGFR change from baseline to 1 year follow-up of patients in the Ketosteril and non-Ketosteril groups. The lower whisker is the first quartile minus 1.5 times the interquartile range and the upper whisker is the third quartile plus the 1.5 times interquartile range.

After 1 year follow-up, the Ketosteril group exhibited a significantly lower incidence of new-onset ESRD requiring maintenance dialysis than the non-Ketosteril group did (40.2% vs. 44.4%, subdistribution hazard ratio (SHR): 0.80, 95% confidence interval (CI): 0.70–0.91). Regarding secondary outcomes, the Ketosteril group exhibited a significantly lower risk of MACCEs than the non-Ketosteril group did (3.7% vs. 5.9%, HR: 0.61, 95% CI: 0.38–0.97). The risk of other time to event outcomes did not significantly differ between the two groups (Table 2). After additionally adjusting for hypertension, diabetes, ACEIs/ARBs, hepatitis C virus infection, calcium supplementation and vitamin D therapy, the results were not altered. The change in renal function between the groups is illustrated in Figure 2.

The eGFR significantly declined in both study groups (Ketosteril group: from 6.7 to 4.7 mL/min/1.73 m^2^; non-Ketosteril group: from 6.7 to 5.2 mL/min/1.73 m^2^) whereas the range of renal function decline did not significantly differ between the groups.

### 3.3. Risk of Dialysis across Different Baseline eGFRs

**Figure 3:**
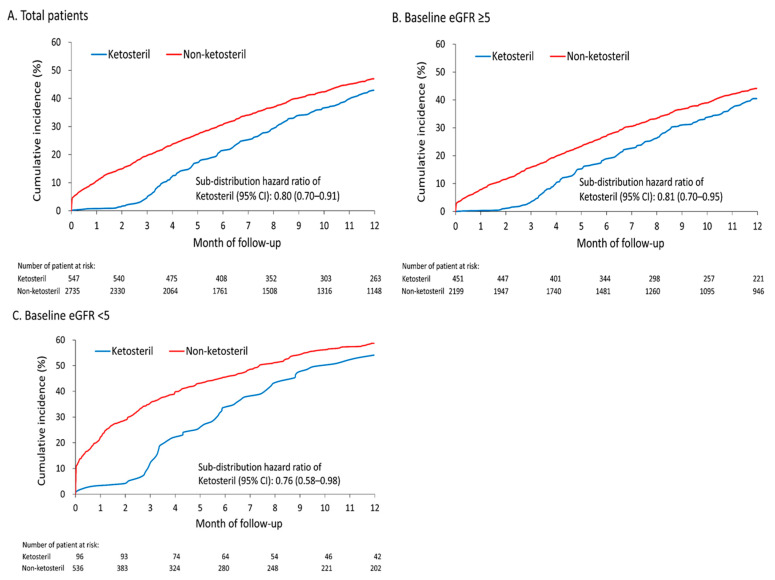
Cumulative incidence function of new-onset ESRD requiring the maintenance dialysis of patients in the Ketosteril and non-Ketosteril groups in the whole study population (**A**); patients with baseline eGFR > 5 mL/min/1.73 m^2^ (**B**); and patients with baseline eGFR < 5 mL/min/1.73 m^2^ (**C**).

As illustrated in Figure 3, to elucidate the effects of sLPD on patients with different baseline renal functions, we analyzed the cumulative incidence of new-onset ESRD requiring maintenance dialysis in the whole study population (Figure 3A), in patients with baseline eGFR > 5 mL/min/1.73 m^2^ (Figure 3B), and in patients with baseline eGFR < 5 mL/min/1.73 m^2^ (Figure 3C).

**Figure 4:**
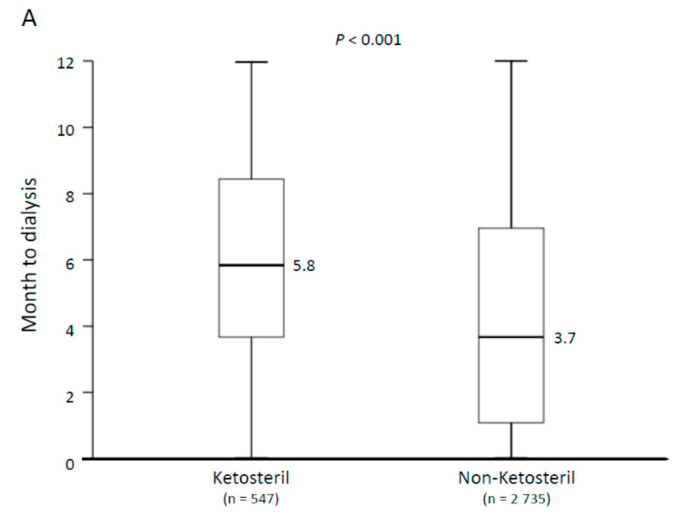

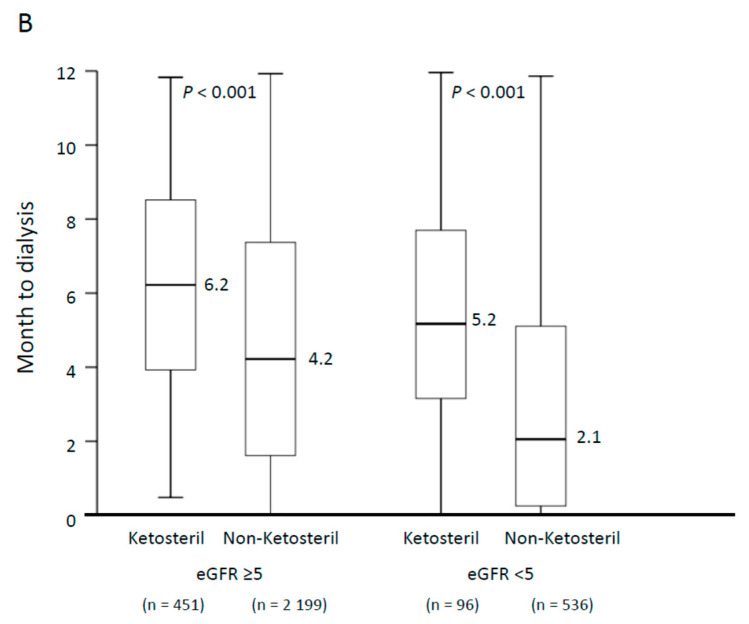
Median duration from the index date to the day of initiating the dialysis of patients in the Ketosteril and non-Ketosteril groups in the whole study population (**A**) and stratified by baseline eGFR level (**B**). The lower whisker is the first quartile minus 1.5 times the interquartile range and the upper whisker is the third quartile plus 1.5 times the interquartile range.

The Ketosteril group exhibited a significantly lower incidence of new-onset maintenance dialysis than the non-Ketosteril group did irrespective of the baseline eGFR level. As shown in Figure 4, the Ketosteril group had a longer duration between the index date and the dialysis date (median: 5.8 months vs. 3.7 months; *p* < 0.001). Irrespective of baseline eGFR, this difference remained significant between the Ketosteril and non-Ketosteril groups. Figure 3 and Figure 4 suggest that the benefit of sLPD treatment in delaying the initiation of maintenance dialysis remained even for patients with eGFR < 5 mL/min/1.73 m^2^.

## 4. Discussion

Numerous randomized controlled trials and meta-analyses have indicated that, among patients who can adhere to dietary therapy and the intensive monitoring of nutritional status with an eGFR 10–30 mL/min/1.73 m^2^, sVLPDs or sLPDs can safely retard CKD progression [19], delay the initiation of dialysis [20,21], and improve several metabolic biomarkers [22,23]. However, less evidence exists regarding whether these beneficial effects and safety remain if the dietary therapy is only started at eGFR < 10 mL/min/1.73 m^2^. The practice of dietary therapy in Taiwan is suitable to evaluate this question because National Health Insurance regulations mandate that a low-protein diet supplemented with Ketosteril is prescribed without the need of co-payment only for patients with creatinine > 6 mg/dL, approximately equal to eGFR 5–10 mL/min/1.73 m^2^. Thus, this study provides some of the first evidence that sLPDs, when started in patients with extremely low eGFR, can still delay the initiation of dialysis without inducing a higher risk of adverse events.

Most patients (85.3%) in this study presented with an eGFR of less than 10 mL/min/1.73 m^2^ when they started an sLPD, and the mean baseline eGFR was 6.7 mL/min/1.73 m^2^, which is much lower than those reported in previous research. For example, MDRD trial-enrolled patients had an eGFR of 13–24 mL/min/1.73 m^2^ [15], whereas Garneata et al. enrolled stable CKD4 patients [24]. The difference in enrolled populations allowed this study to focus on those with extremely low baseline renal function, who have been less examined in previous research. By assigning five patients without receiving any dietary therapy (non-sLPD group) to one treated patient (sLPD group) from the same dataset with matched age, sex, and baseline eGFR, this study simulated a randomized control trial to evaluate the effect of sLPD treatment. After 1 year follow-up, this study found that sLPD treatment could delay the initiation of dialysis for an average of 2.1 months. Notably, the benefit of an sLPD in delaying dialysis remained the same for patients with a baseline eGFR ≥ 5 mL/min/1.73 m^2^ or < 5 mL/min/1.73 m^2^. This suggests that dietary therapy is still effective even for a few weeks or months before renal replacement therapy is required. Similarly, in an RCT that enrolled 112 patients older than 70 years and near dialysis (eGFR 4–6 mL/min/1.73 m^2^), Brunori et al. showed that the initiation of dialysis could be safely delayed in the sVLPDs arm compared to those in dialysis arm [25]. Our results suggest that the findings of Brunori et al. may not be limited to elderly patients and may hold true for the whole severe CKD population. However, unlike Garneata et al. [24], who reported that very strict dietary therapy among vegetarians with moderate to advanced CKD could both retard the decline of eGFR and improve metabolic abnormalities, our study did not find similar benefits. Accordingly, we speculate that the benefit of dietary therapy among these patients with extremely poor renal function is mainly attributable to the reduction of the generation of uremic toxins rather than the direct slowing of nephron loss.

Patients with extremely poor renal function are a fragile population with the high rates of malnutrition, infection, and cardiovascular disease [3,26,27]. The safety of dietary therapy while treating these patients is another important issue.

In line with a previous RCT [12] and observational study [28], this study exhibited that, after 1 year follow-up, dietary therapy did not increase the probability of all-cause mortality and infection, and that it slightly reduced MACCEs. Two previous studies [10,14] have indicated that dietary therapy could reduce the occurrence of MACCEs after dialysis initiation, and the results of the current study further suggests that the benefit may directly start with the initiation of dietary therapy. Many previous studies have demonstrated that sLPD or sVLPD could reduce dyslipidemia [29], hypertension [30], calcium/phosphate imbalance [28], and proteinuria [31], which are all regarded as important risk factors of cardiovascular diseases among CKD patients. Thus, we speculated that the benefit of dietary therapy in reducing MACCEs is via the reduction of these metabolic imbalance.

Several limitations of this study should be acknowledged. First, despite the design of this study seeking to eliminate confounders, observational studies entail some inherent bias. Second, although there are strict regulations governing nutritional counseling and monitoring during sLPD treatment in the Chang Gung Memorial Hospital network, patient adherence to a low protein diet cannot be simply ascertained through a database study. Third, some clinical characteristics, namely systolic blood pressure, diastolic blood pressure, and body mass index, are not available in the database. Finally, this study was performed based on the dataset of a hospital’s network in Taiwan; thus, the applicability of these results to wider populations is unknown.

In conclusion, this study found that, among patients with extremely low baseline GFR, sLPD treatment safely delayed the need for dialysis without increasing the rates of malnutrition, MACCEs, or mortality. By delaying dialysis, patients were better able to prepare dialysis access or complete pre-transplantation evaluation, which could represent considerable savings in medical expenses for the health care system. Accordingly, although this study demonstrates that sLPD treatment may only postpone rather than prevent dialysis, it still indicates that dietary therapy is worth initiating even among patients with extremely low GFR.

## Figures and Tables

**Table 1:** Baseline characteristics of patients with advanced CKD in the Ketosteril and non-Ketosteril groups.

Parameter	Valid *N*	Ketosteril Group (*n* = 547)	Non- Ketosteril Group (*n* = 2735)	*p* Value
Male sex	3282	278 (50.8)	1383 (50.6)	0.913
Age (years)	3282	62.2 (53.3, 72.0)	62.7 (53.7, 71.7)	0.777
Baseline comorbidity				
Diabetes	3282	224 (41.0)	1122 (41.0)	0.975
Hepatitis B virus infection	3282	5 (0.9)	10 (0.4)	0.083
Hepatitis C virus infection	3282	6 (1.1)	3 (0.1)	<0.001
Hypertension	3282	366 (66.9)	1758 (64.3)	0.240
Renal function				
eGFR (mL/min/1.73 m^2^)	3282	6.7 (5.4, 8.5)	6.7 (5.3, 8.8)	0.830
Baseline eGFR < 10 (%)	3282	477 (87.2)	2322 (84.9)	0.165
Baseline eGFR < 5 (%)	3282	96 (17.6)	536 (19.6)	0.268
Albumin/creatinine ratio (mg/d)	152	2072 (1106, 3448)	1857 (575, 4029)	0.842
Urine protein (U)/creatinine ratio (mg/d)	556	2053 (1104, 4987)	2688 (1046, 5640)	0.440
Laboratory data				
HbA1c (%)	1420	6.2 (5.6, 7.0)	6.3 (5.7, 7.2)	0.174
Total cholesterol (mg/dL)	1674	172 (146, 196)	170 (144, 199)	0.556
Triglyceride, mg/dL	1652	118 (83, 185)	123 (86, 181)	0.692
Antihypertensive therapy				
Antihypertensive drugs	3282	366 (66.9)	1758 (64.3)	0.240
ACEIs/ARBs	3282	324 (59.2)	1318 (48.2)	<0.001
Nitrogen waste products				
Serum urea (mg/dL)	3055	77.6 (62.0, 93.5)	74.0 (58.0, 93.0)	0.022
Serum uric acid (mg/dL)	2036	7.6 (6.6, 8.8)	7.5 (6.4, 8.9)	0.434
Acid-base balance				
Serum bicarbonate (mEq/L)	1542	19.5 (17.0, 22.0)	20.4 (17.6, 22.8)	0.008
Calcium-phosphorus metabolism				
Serum calcium (mg/dL)	2839	8.6 (8.2, 8.9)	8.6 (8.1, 9.1)	0.106
Serum phosphates (mg/dL)	2725	5.1 (4.3, 5.8)	5.1 (4.3, 5.9)	0.951
Calcium supplementation	3282	145 (26.5)	566 (20.7)	0.003
Vitamin D therapy	3282	62 (11.3)	174 (6.4)	<0.001

Abbreviations: ACEIs, angiotensin converting enzyme inhibitors; ARBs, angiotensin II receptor blockers; CKD, chronic kidney injury; eGFR, estimated glomerular filtration rate; HbA1c, glycated hemoglobin. Data are given as the median (25th and 75th percentile) or frequency (percentage).

**Table 2:** Clinical outcomes at 1 year of patients with advanced CKD in the Ketosteril and non-Ketosteril groups.

			Unadjusted Analysis		Adjusted Analysis #	
Outcome	Ketosteril Group (*n* = 547)	Non-Ketosteril Group (*n* = 2735)	HR or SHR of Ketosteril (95% CI)	*p* Value	HR or SHR of Ketosteril (95% CI)	*p* Value
Primary outcome: dialysis	220 (40.2)	1215 (44.4)	0.80 (0.70–0.91)	0.001	0.73 (0.64–0.84)	<0.001
Secondary outcome:						
All-cause mortality	10 (1.8)	67 (2.4)	0.73 (0.38–1.43)	0.362	0.74 (0.38–1.43)	0.367
Acute myocardial infarction	7 (1.3)	63 (2.3)	0.55 (0.25–1.19)	0.129	0.50 (0.23–1.11)	0.088
Ischemic stroke	6 (1.1)	46 (1.7)	0.64 (0.28–1.50)	0.309	0.61 (0.26–1.42)	0.253
MACCE *	20 (3.7)	160 (5.9)	0.61 (0.38–0.97)	0.035	0.58 (0.36–0.92)	0.021
Infection-related hospitalization	85 (15.5)	479 (17.5)	0.86 (0.68–1.08)	0.193	0.83 (0.66–1.05)	0.126
Heart failure hospitalization	15 (2.7)	95 (3.5)	0.78 (0.45–1.34)	0.362	0.73 (0.42–1.25)	0.247

Abbreviations: CI, confidence interval; HR, hazard ratio; MACCE, major adverse cardiac and cerebrovascular events; SHR, subdistribution hazard ratio. * All-cause mortality, acute myocardial infarction, or ischemic stroke. # Adjusted for hypertension, diabetes, ACEIs/ARBs, hepatitis C virus infection, calcium supplementation and vitamin D therapy; data are given as a frequency (percentage).

## References

[1] Addis T., Lew W. (1939). Diet and Death in Acute Uremia. J. Clin. Investig..

[2] Lewis D.S. (1921). On the Influence of a Diet with High Protein Content on the Kidney. Can. Med. Assoc. J..

[3] Cuppari L., Meireles M.S., Ramos C.I., Kamimura M.A. (2014). Subjective Global Assessment for the Diagnosis of Protein–Energy Wasting in Nondialysis-Dependent Chronic Kidney Disease Patients. J. Ren. Nutr..

[4] Walser M. (1975). Ketoacids in the treatment of uremia. Clin. Nephrol..

[5] Kang C.W., Tungsanga K., Walser M. (1986). Effect of the level of dietary protein on the utilization of alpha-ketoisocaproate for protein synthesis. Am. J. Clin. Nutr..

[6] Sunil P., Pande D.P., Sharma S., Sharma D., Bal C.S., Kulkarni H. (2004). Randomized, double-blind, placebo-controlled trial to evaluate efficacy of ketodiet in predialytic chronic renal failure. J. Ren. Nutr..

[7] National Kidney Foundation Kidney Disease Outcomes Quality Initiative (2000). Clinical practice guidelines for nutrition in chronic renal failure. Am. J. Kidney Dis..

[8] Walser M., Hill S.B., Ward L., Magder L. (1993). A crossover comparison of progression of chronic renal failure: Ketoacids versus amino acids. Kidney Int..

[9] Jiang N., Qian J., Sun W., Lin A., Cao L., Wang Q., Ni Z., Wan Y., Linholm B., Axelsson J. (2009). Better preservation of residual renal function in peritoneal dialysis patients treated with a low-protein diet supplemented with keto acids: A prospective, randomized trial. Nephrol. Dial. Transplant..

[10] Bellizzi V., Chiodini P., Cupisti A., Viola B.F., Pezzotta M., De Nicola L., Minutolo R., Barsotti G., Piccoli G.B., Di Iorio B. (2015). Very low-protein diet plus ketoacids in chronic kidney disease and risk of death during end-stage renal disease: A historical cohort controlled study. Nephrol. Dial. Transplant..

[11] Wu C.-H., Yang Y.-W., Hung S.-C., Kuo K.-L., Wu K.-D., Wu V.-C., Hsieh T.-C., National Taiwan University Study Group on Acute Renal Failure (NSARF) (2017). Ketoanalogues supplementation decreases dialysis and mortality risk in patients with anemic advanced chronic kidney disease. PLoS ONE.

[12] Cianciaruso B., Pota A., Pisani A., Torraca S., Annecchini R., Lombardi P., Capuano A., Nazzaro P., Bellizzi V., Sabbatini M. (2008). Metabolic effects of two low protein diets in chronic kidney disease stage 4-5--a randomized controlled trial. Nephrol. Dial. Transplant..

[13] Piccoli G.B., Nazha M., Capizzi I., Vigotti F.N., Mongilardi E., Bilocati M., Avagnina P., Versino E. (2016). Patient Survival and Costs on Moderately Restricted Low-Protein Diets in Advanced CKD: Equivalent Survival at Lower Costs?. Nutrients.

[14] Yen C.-L., Tu K.-H., Lin M.-S., Chang S.-W., Fan P.-C., Hsiao C.-C., Chen C.-Y., Hsu H.-H., Tian Y.-C., Chang C.-H. (2018). Does a Supplemental Low-Protein Diet Decrease Mortality and Adverse Events After Commencing Dialysis? A Nationwide Cohort Study. Nutrients.

[15] Klahr S., Levey A.S., Beck G.J., Caggiula A.W., Hunsicker L., Kusek J.W., Striker G. (1994). The Effects of Dietary Protein Restriction and Blood-Pressure Control on the Progression of Chronic Renal Disease. N. Engl. J. Med..

[16] Cianciaruso B., Capuano A., D’Amaro E., Ferrara N., Nastasi A., Conte G., Bellizzi V., Andreucci V.E. (1989). Dietary compliance to a low protein and phosphate diet in patients with chronic renal failure. Kidney Int. Suppl..

[17] Tsai M.-S., Lin M.-H., Lee C.-P., Yang Y.-H., Chen W.-C., Chang G.-H., Tsai Y.-T., Chen P.-C., Tsai Y.-H. (2017). Chang Gung Research Database: A multi-institutional database consisting of original medical records. Biomed. J..

[18] Shao S., Chan Y., Yang Y.K., Lin S., Hung M., Chien R., Lai C., Lai E.C. (2019). The Chang Gung Research Database—A multi-institutional electronic medical records database for real-world epidemiological studies in Taiwan. Pharmacoepidemiol. Drug Saf..

[19] Kasiske B.L., Lakatua J.D., Ma J.Z., Louis T.A. (1998). A meta-analysis of the effects of dietary protein restriction on the rate of decline in renal function. Am. J. Kidney Dis..

[20] Laville M., Boissel J.-P. (2006). Low protein diets for chronic kidney disease in non diabetic adults. Cochrane Database Syst. Rev..

[21] Pedrini M.T., Levey A.S., Lau J., Chalmers T.C., Wang P.H. (1996). The Effect of Dietary Protein Restriction on the Progression of Diabetic and Nondiabetic Renal Diseases. Ann. Intern. Med..

[22] Malvy D., Maingourd C., Pengloan J., Bagros P., Nivet H. (1999). Effects of severe protein restriction with ketoanalogues in advanced renal failure. J. Am. Coll. Nutr..

[23] Di Iorio B., Di Micco L., Torraca S., Sirico M.L., Russo L., Pota A., Mirenghi F., Russo D. (2012). Acute Effects of Very-Low-Protein Diet on FGF23 Levels: A Randomized Study. Clin. J. Am. Soc. Nephrol..

[24] Garneata L., Stancu A., Dragomir D., Stefan G., Mircescu G. (2016). Ketoanalogue-Supplemented Vegetarian Very Low–Protein Diet and CKD Progression. J. Am. Soc. Nephrol..

[25] Brunori G., Viola B.F., Parrinello G., De Biase V., Como G., Franco V., Garibotto G., Zubani R., Cancarini G.C. (2007). Efficacy and Safety of a Very-Low-Protein Diet When Postponing Dialysis in the Elderly: A Prospective Randomized Multicenter Controlled Study. Am. J. Kidney Dis..

[26] Menon V., Gul A., Sarnak M.J. (2005). Cardiovascular risk factors in chronic kidney disease. Kidney Int..

[27] Hassan H.I.C., Tang M., Djurdjev O., Langsford D., Sood M.M., Levin A. (2016). Infection in advanced chronic kidney disease leads to increased risk of cardiovascular events, end-stage kidney disease and mortality. Kidney Int..

[28] Mircescu G., Gârneaţă L., Stancu S., Capuşă C. (2007). Effects of a Supplemented Hypoproteic Diet in Chronic Kidney Disease. J. Ren. Nutr..

[29] Bernard S., Fouque D., Laville M., Zech P. (1996). Effects of low-protein diet supplemented with ketoacids on plasma lipids in adult chronic renal failure. Miner. Electrolyte Metab..

[30] Bellizzi V., Di Iorio B., De Nicola L., Minutolo R., Zamboli P., Trucillo P., Catapano F., Di Cristofano C., Scalfi L., Conte G. (2007). Very low protein diet supplemented with ketoanalogs improves blood pressure control in chronic kidney disease. Kidney Int..

[31] Chauveau P., Combe C., Rigalleau V., Vendrely B., Aparicio M. (2007). Restricted Protein Diet Is Associated With Decrease in Proteinuria: Consequences on the Progression of Renal Failure. J. Ren. Nutr..

